# Systemic Delivery of a Dual PI3K/mTOR Inhibitor More Effective than Topical Delivery in Preventing Anal Carcinogenesis in an HPV Transgenic Mouse Model

**DOI:** 10.26502/jcsct.5079153

**Published:** 2022-04-18

**Authors:** Laura C Gunder, Tyra H Moyer, Marissa R Ziolkowski, Margaret K Keating, Glen E Leverson, Wei Zhang, Evie H Carchman

**Affiliations:** 1 Department of Surgery, University of Wisconsin-Madison, Madison, Wisconsin, USA; 2 Waisman Center, University of Wisconsin-Madison, Madison, Wisconsin, USA; 3 Department of Cell and Regenerative Biology, University of Wisconsin-Madison, Madison, Wisconsin, USA; 4 Department of Pathology and Laboratory Medicine, University of Wisconsin-Madison, Madison, Wisconsin, USA; 5 William S. Middleton Memorial Veterans Hospital, Madison, Wisconsin, USA; 6 University of Wisconsin Carbone Cancer Center, UW Health University Hospital, Madison, Wisconsin, USA

**Keywords:** Anal dysplasia, Anal cancer, Dual PI3K/mTOR inhibitor, LY3023414, Squamous cell carcinoma of the anus

## Abstract

**Introduction:**

Anal dysplasia is a growing health concern that over time can result in squamous cell carcinoma (SqCC) of the anus. In this study, we compare a topical versus systemic (oral) administration of LY3023414, a dual PI3K/mTOR inhibitor, to prevent anal carcinogenesis in a Human Papillomavirus (HPV) mouse model of anal cancer.

**Materials and Methods:**

*K14E6/E7* transgenic mice were used to model HPV-induced anal carcinogenesis. Mice with varying starting anal histologies (normal histology, low-grade, and high-grade anal dysplasia) were treated topically at the anus or systemically via oral gavage with LY3023414 with or without topical carcinogen for 20 weeks. Mice were monitored for overt anal tumor development and anal tissue was assessed for histology and markers of PI3K and mTOR activity (pAKT and pS6, respectively).

**Results:**

LY3023414 treatment, regardless of the mode of delivery, significantly decreased overt tumor development in mice starting with normal histology and low-grade anal dysplasia. Systemic LY3023414 treatment was more effective in delaying tumor onset than topical treatment. Mice treated with systemic LY3023414 had significantly reduced rates of anal SqCC when starting with normal and low-grade anal dysplasia compared to topical treatment. Topical treatment was only effective in reducing SqCC in the setting of low-grade dysplasia. LY3023414 inhibition of pAKT and pS6 expression varied with starting histology. Neither treatment mode was effective in the setting of high-grade anal dysplasia.

**Conclusion:**

Systemic LY3023414 treatment was more effective than topical application in delaying the progression of normal anal histology and low-grade dysplasia to anal cancer in HPV-associated mice.

## 1. Introduction

Anal cancer is a growing health concern due to its rising incidence and mortality [1]. While anal cancer is considered a rare cancer, it is estimated that in the United States alone, roughly 9,000 new cases of anal cancer will be diagnosed, and 1,400 people will die from the disease in 2022 [1]. The majority (91%) of all cases are Human Papillomavirus (HPV) positive with 87% of cases due to high-risk strains HPV16 and HPV18 [2–4]. Anal dysplasia is the precursor lesion to anal cancer and is common in immunocompromised individuals, such as Human Immunodeficiency Virus (HIV)-positive patients [5–7]. Current treatments for anal dysplasia that aim to prevent anal cancer are ineffective and thus, there is a need for the identification of novel therapies to treat anal dysplasia to prevent anal cancer. There is currently no standard of care topical treatment for anal dysplasia, since all current treatments are off-label options [8, 9]. Commonly used therapies to treat anal dysplasia are ablative techniques and topical treatments [8]. Ablative techniques include the removal or destruction of precancerous lesions, which can have acute and chronic side effects [10]. Topical therapies include 5-fluorouracil (5-FU) and Imiquimod. Topical 5-FU works to prevent cell proliferation and promote cell death [8, 10], while Imiquimod induces apoptosis at the treatment site [9,11]. Neither of these topical treatments *specifically* targets dysplastic cells or those cells infected with HPV, and therefore both cause local irritation, erythema, and ulceration to normal tissue [8, 9]. Beyond these challenges, the aforementioned treatments are not efficacious long-term, with associated recurrence rates over 50% [10]. Therefore, identifying treatments (topical or systemic) that minimize discomfort and maximize benefit is necessary, and seeking treatments that target HPV-affected cells is ideal.

Infection with high-risk HPV involves the expression of oncoproteins E6 and E7. When these viral oncoproteins are expressed, there is dysregulation of cellular pathways such as the phosphatidylinositol 3-kinase (PI3K) and mechanistic target of rapamycin (mTOR), leading to decreased apoptosis and increased cell proliferation, cellular migration, and invasion ([12, 13, 14]. The PI3K and mTOR pathways are dysregulated in many cancers, including anal cancer [15, 16]. PI3K is divided into 3 classes, of which class I PI3K isoforms are often implicated in cancer development [17–19]. mTOR is a kinase downstream of PI3K and is made up of two catalytic subunits, mTORC1, and mTORC2. [19–21]. Both PI3K and mTOR direct a myriad of cellular processes, including cellular proliferation, growth, and survival. Dual PI3K/mTOR inhibitors that inhibit PI3K class I isoforms, mTORC1, and mTORC2, have shown promise in cancer-related clinical trials [17, 19, 22, 23]. It has been previously shown that the topical administration of BEZ235, a dual PI3K/mTOR pathway inhibitor, prevents anal carcinogenesis in a mouse model of HPV-associated anal cancer [24]. These *K14E6/E7* mice are bred to express HPV genotype 16 oncoproteins E6 and E7 in their epithelium and spontaneously develop anal dysplasia comparable to HPV-positive patients. In this study, we compare the topical versus oral (systemic) administration of LY3023414, a dual PI3K/mTOR inhibitor, in order to slow the progression of anal dysplasia to anal cancer.

## 2. Methods

### 2.1 Mice

All mice were maintained in an American Association for Accreditation of Laboratory Animal Care-approved Wisconsin Institute for Medical Research (WIMR) Animal Care Facility. The experiments were performed in accordance and with the approval of the Institutional Animal Care and Use Committee (M005946). *K14E6/E7* transgenic mice were generated as previously described in Stelzer et al. [25]. Equal numbers of male and female mice were divided into groups based on starting histology: 5 weeks of age, when the majority (>75%) of transgenic mice maintain normal anal histology; 15 weeks of age, when the majority of mice display low-grade anal dysplasia; and 25 weeks of age, when the majority of mice develop high-grade anal dysplasia [26]. Mice were then randomized into treatment groups within each starting histology: control (no treatment), topical DMBA alone, topical LY3023414 alone, systemic LY3023414 alone, topical LY3023414 + topical DMBA, and systemic LY3023414 + topical DMBA. Mice were monitored daily for local side effects and thoroughly examined weekly for the development of overt anal tumors. Mice were sacrificed 20 weeks after initiation of treatment or if their tumors measured or exceeded 20×20mm.

### 2.2 Carcinogen Treatment

In order to ensure anal carcinogenesis, mice were treated topically at the anus with the carcinogen 7, 12 dimethylbenz [a] anthracene (DMBA, Cat. No. D3254, Sigma Aldrich, Saint Louis, MO, USA). We have previously shown that treating these transgenic mice for 20 weeks with a topical DMBA solution can result in 100% development of SqCC of the anus (Carchman et al. 2016). Mice were dosed topically at the anus, once weekly with a 20 μL solution of 0.12 μmol DMBA in a 60% acetone (CAS No. 67-64-1, Avantor, Radnor, PA, USA) and 40% dimethyl sulfoxide (DMSO, Cat. No. BP231–1, Thermo Fisher Scientific, Waltham, MA, USA). Protocol was adapted from Stelzer et al..

### 2.3 Topical LY3023414 Treatment

To determine the topical dose of LY3023414 (Cat. No.: HY-12513, MedChem Express, Monmouth Junction, NJ, USA), four different concentrations, 0.5, 1.0, 2.5, and 5.0%, of LY3023414, dissolved in polyethylene glycol (PEG-300, Cat. No. 202371, Sigma Aldrich, Saint Louis, MO, USA), were applied topically to the anus of mice (N=3 per group) for two consecutive weeks, five days a week. 1% LY3023414 was the lowest concentration that resulted in a decrease in pAKT and pS6 expression, markers of the PI3K and mTOR activity respectively, as assessed by immunohistochemical staining (data not shown). Subsequently, all mice receiving topical LY3023414 were treated five days a week, Monday-Friday, topically at the anus with 20 μL of 1% LY3023414 in PEG-300.

### 2.4 Systemic LY3023414 Treatment

LY3023414 was dissolved in DMSO and 1% hydroxyethyl cellulose (Sigma Aldrich, Saint Louis, MO, USA) in sterile water. Mice were treated five days a week, Monday-Friday, via oral gavage with 4.5mg/kg of body weight per day. Protocol was adapted from Smith et al. [27].

### 2.5 Histology

After sacrifice, anal tissue was collected and fixed in 4% paraformaldehyde solution (Cat. No. J19943-K2, Thermo Fisher Scientific, Waltham, MA, USA) for 24 hours and then placed in 70% ethanol. After fixation, the tissue was bisected and processed for sectioning. The tissue was embedded in paraffin and serially sectioned at 5 μm thickness. Every fifth section was stained with hematoxylin and eosin (H&E) by the University of Wisconsin Carbone Cancer Center Experimental Animal Pathology Laboratory and histology evaluated by a trained Gastrointestinal Pathologist for evidence of dysplasia or invasive squamous cell carcinoma (SqCC).

### 2.6 Immunohistochemistry

Paraffin-embedded tissue sections were deparaffinized, rehydrated, subjected to citrate buffer antigen retrieval, permeabilized with 2N HCl, and then blocked with 5% horse serum. Sections were then stained with a monoclonal rabbit antibody against pAKT serine 473 (1:50 in 5% horse serum in PBS, antibody #3787; Cell Signaling Technology, Danvers, Massachusetts, USA) or a polyclonal rabbit antibody against pS6 ribosomal protein Ser235/236 (1:50 in 5% horse serum in PBS, antibody #2211; Cell Signaling Technology, Danvers, MA, USA) overnight at 4°C. Each slide was then washed and incubated with VectaStain universal secondary antibody (1:50 in 5% horse serum in PBS; Vector Laboratories, Inc., Burlingame, California, USA). Slides were then subjected to R.T.U. VectaStain ABC reagent (Vector Laboratories, Inc., Burlingame, California, USA). Finally, sections were incubated with 3,3′-diaminobenzidine substrate (DAB Peroxidase Substrate; Vector Laboratories, Inc., Burlingame, California, USA); counterstained with hematoxylin (Hematoxylin QS, Vector Laboratories, Inc., Burlingame, California, USA); dehydrated; and mounted. Light microscopy was performed and images at ×200 magnification were acquired using the Zeiss Axio Imager M2 imaging system. Images were then analyzed via ImageJ with measurements taken in RawIntDen/Area (raw integrated density/area) of the intensity signal.

### 2.7 Statistical analysis

To detect at least a 50% difference in tumor incidence with a type I error rate of 5% and a type II error rate of 20% (80% power) between the treated and untreated groups, 12 mice per group were needed. ANOVA or Fisher’s two-sided exact t-tests were used to determine differences between treatment groups in overt anal tumor incidence after 20 weeks of treatment. Kaplan–Meier analysis with log-rank testing was applied to determine differences in time to tumor onset. Fisher’s two-sided exact t-tests were used to determine significance between treatment groups and histological outcome of SqCC or no SqCC. The comparisons between treatment groups and expression of pAKT and pS6 were made using a one-way analysis of variance with Tukey post-hoc testing. SPSS, version 24 (IBM, Armonk/North Castle, NY, USA) was utilized to perform each of these analyses. Statistical significance was defined as a p-value of 0.05 or less.

## 3. Results

### 3.1 Mice Treated systemically or topically with LY3023414 Developed Less Overt Anal Tumors when starting with 3.1.1 Normal anal histology or low-grade anal dysplasia

#### 3.1.1.1 Normal histology/5 weeks of age

**Figure 1:**
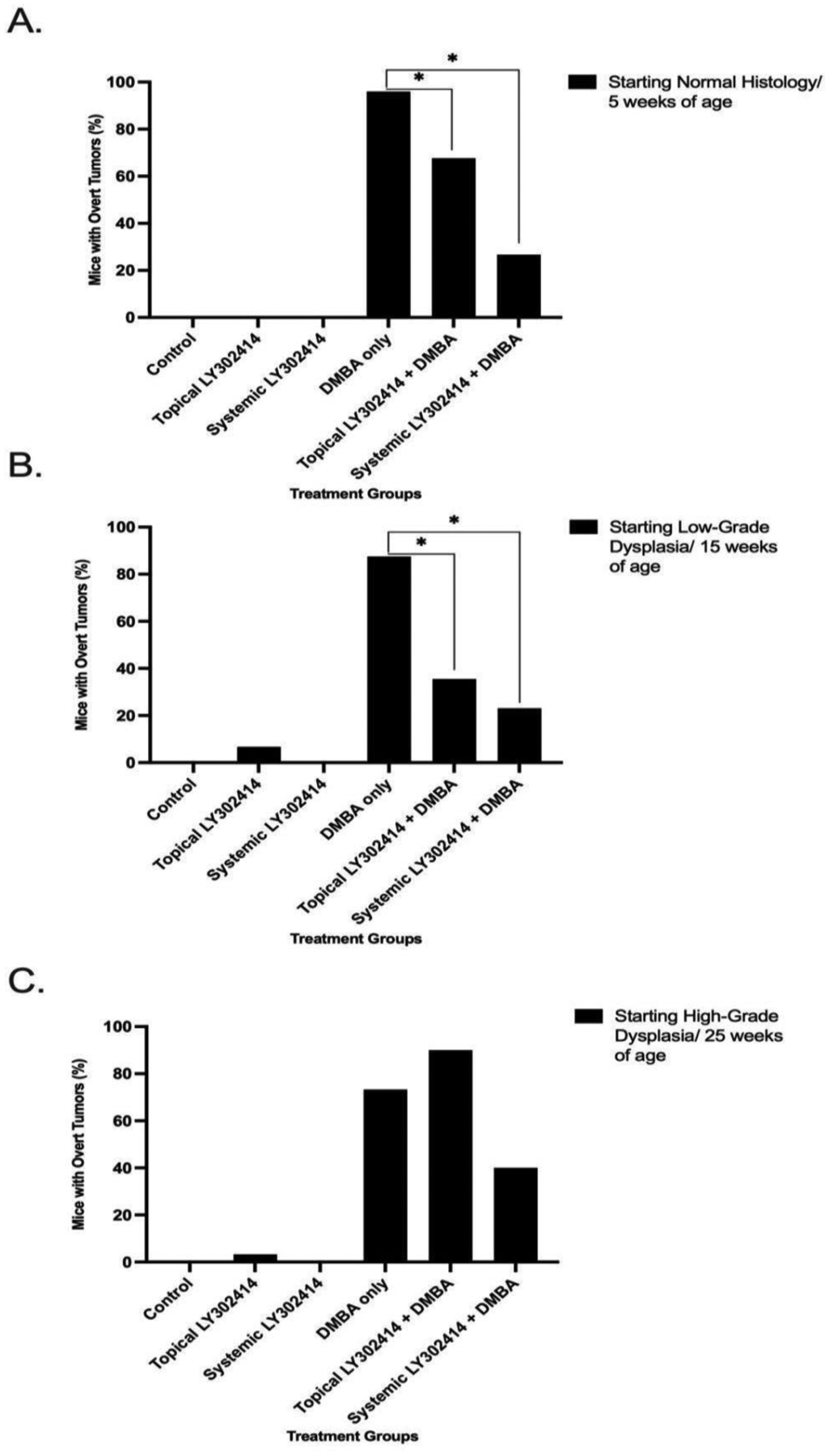
The percentage of K14E6/E7 mice in each treatment group that developed overt anal tumors at any point during the 20-week period treatment. The notation * represents statistical significance (p <0.05) between groups. **A.** Mice that began treatment at 5 weeks of age/ normal anal histology. **B.** Mice that began treatment at 15 weeks of age/ low-grade anal dysplasia. **C.** Mice that began treatment at 25 weeks of age/ high-grade anal dysplasia.

None of the mice in the control (0/30), topical LY3023414 only (0/30), or systemic LY3023414 only (0/14) groups developed overt anal tumors at any point during the 20-week treatment period. Almost all of the mice, 96% (24/25), that were treated with topical DMBA alone developed overt anal tumors. Only 67.7% (21/31) of the mice treated with topical LY3023414 + topical DMBA and only 26.7% (4/15) of systemic LY3023414 + topical DMBA developed anal tumors. There was a statistically significant reduction in the number of overt tumors that mice developed within the 20 weeks for both topical LY3023414 + topical DMBA and systemic LY3023414 + topical DMBA groups as compared to the topical DMBA-only group (p-values = 0.0151 and < 0.0001, respectively) (Figure 1A).

#### 3.1.1.2 Low-Grade Anal Dysplasia/15 weeks of age

None of the control (0/30) or systemic LY3023414-only (0/12) mice developed tumors, and 6.67% (2/30) of the topical LY3023414-only mice developed overt tumors. For topical LY3023414 + topical DMBA mice, 35.5% (11/31), and systemic LY3023414 + topical DMBA, 23% (3/13), developed anal tumors as compared to 87.5% (28/32) of the topical DMBA only treated mice. There was a statistically significant reduction in the number of overt tumors that mice developed in both topical LY3023414 + topical DMBA and systemic LY3023414 + topical DMBA as compared to topical DMBA-only mice (p-values < 0.0001) (Figure 1B).

#### 3.1.1.3 High-Grade Anal Dysplasia/25 weeks of age

A few of the drug-only mice developed tumors: 6.67% (2/30) of the control mice, 3.33% (1/30) of the topical LY3023414-only mice, and none (0/11) of the systemic LY3023414 mice. A total of 70% (21/30) of the mice treated with topical DMBA alone developed anal tumors as compared to 90% (27/30) of the mice treated with topical LY3023414 + topical DMBA and 40% (4/10) of mice given systemic LY3023414 + topical DMBA. There were no statistically significant reductions in the number of overt tumors that mice developed in either the topical LY3023414 + topical DMBA or the systemic LY3023414 + topical DMBA treatment groups as compared to the topical DMBA- only group (p-values = 0.1806 and 0.1226, respectively) (Figure 1C).

### 3.2 Topical and systemic ly3023414 treatment delayed tumor development in mice starting with normal anal histology or low-grade anal dysplasia

#### 3.2.1 Normal Histology/5 weeks of age

**Figure 2:**
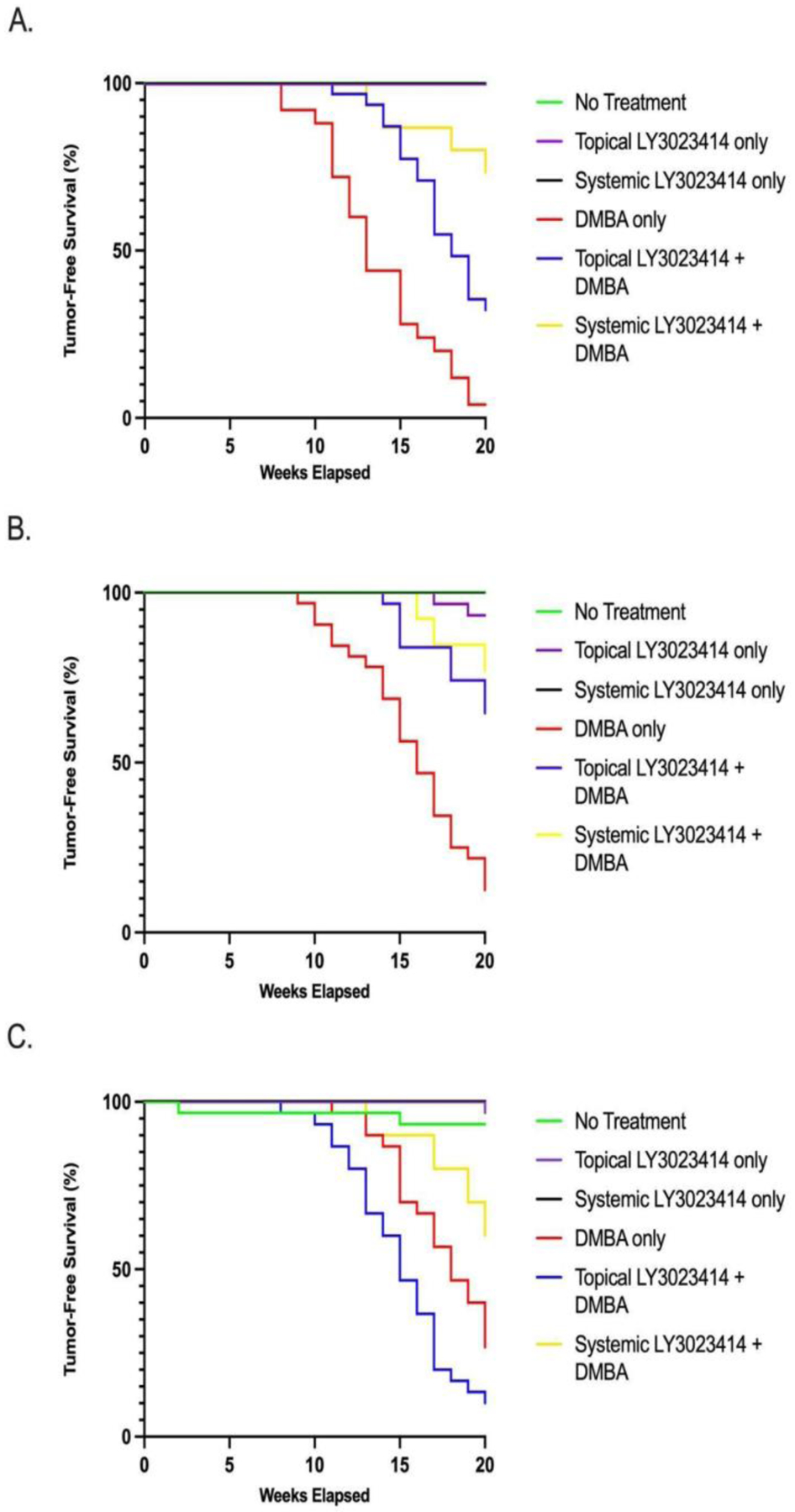
Tumor-free survival as assessed by Kaplan Meyer survival analysis over the 20-week treatment period. A. Mice that began treatment at 5 weeks of age/ normal anal histology. The green-purple-black line shows where no treatment, topical LY3023414-only, and systemic LY3023414-only groups overlap. Statistical significance (p <0.05) was achieved between groups: DMBA-only and systemic LY3023414 + DMBA (red and yellow lines, respectively) and DMBA-only and topical LY3023414 + DMBA groups (red and blue lines, respectively). B. Mice that began treatment at 15 weeks of age/ low-grade anal dysplasia. The green-black line shows where no treatment and systemic LY3023414-only groups overlap. Statistical significance (p <0.05) was achieved between groups: DMBA-only and systemic LY3023414 + DMBA (red and yellow lines, respectively) and DMBA-only and topical LY3023414 + DMBA groups (red and blue lines, respectively) **C.** Mice that began treatment at 25 weeks of age/ high-grade anal dysplasia. The purple-black line shows where topical LY3023414-only and systemic LY3023414-only groups overlap.

Of the 24 out of 25 topical DMBA-treated mice that developed tumors, the average time from treatment commencement to tumor onset was 13.5 weeks ± 3.2 weeks. Groups of mice that developed tumors in the topical LY3023414 + topical DMBA group (21/31) and systemic LY3023414 + topical DMBA mice (4/15), tended to develop tumors later, on average 16.5 ± 2.3 weeks and 16.3 ± 3.3 weeks into the treatment period. Both LY3023414 treatment application modes, topical and systemic, given with topical DMBA significantly increased tumor-free survival as compared to application of topical DMBA-alone (p-values = 0.0421 and 0.0003, respectively) (Figure 2A).

#### 3.2.2 Low-Grade Anal Dysplasia/15 weeks of age

Two of the topical LY3023414-only mice developed overt anal tumors at week 17 and week 19. The average time from treatment commencement to initial tumor onset was 15.25 ± 3.3 weeks for the 28 of 32 mice that developed tumors when given topical DMBA alone. For the mice given topical LY3023414 + topical DMBA, 11/31 mice developed overt tumors at an average of 17.1 ± 2.3 weeks into the treatment period. For the systemic LY3023414 + topical DMBA group, 3/13 mice developed tumors in an average of 17.7± 2.1 weeks into the 20-week treatment period. Both LY3023414 delivery methods, topical and systemic, significantly increased tumor-free survival as compared to DMBA-only treatment (p-value = 0.001 and p-value = 0.0016, respectively) (Figure 2B).

#### 3.2.3 High-Grade Anal Dysplasia/25 weeks of age

One topical LY3023414-only mouse developed an overt anal tumor at the 20-week treatment mark and two control mice developed tumors at week 2 and week 18. For topical DMBA-only mice, 22/30 developed tumors at an average of 16.6 ± 2.3 weeks into the 20-week treatment period. For the topical LY3023414 + topical DMBA, 27/30 mice tended to develop tumors sooner than the topical DMBA-only mice, averaging at 14.6 ± 2.8 weeks into treatment (p-value = 0.2102). Systemic LY3023414 + topical DMBA treatment minimally delayed tumor onset with an average of 17.3 ± 3.1 weeks for the 4/10 mice with tumors as compared to treatment with topical DMBA alone (p-value = 0.2045). None of the LY3023414 treatment modes with DMBA significantly increased tumor-free survival in mice compared to treatment with DMBA alone (Figure 2C).

### 3.3 Mice Treated with Systemic LY3023414 had Less Histologically-Proven SqCC when Starting with Normal Anal 3.3.1. Histology or Low-Grade Anal Dysplasia

#### 3.3.1.1. Normal Histology/5 weeks of age

**Figure 3:**
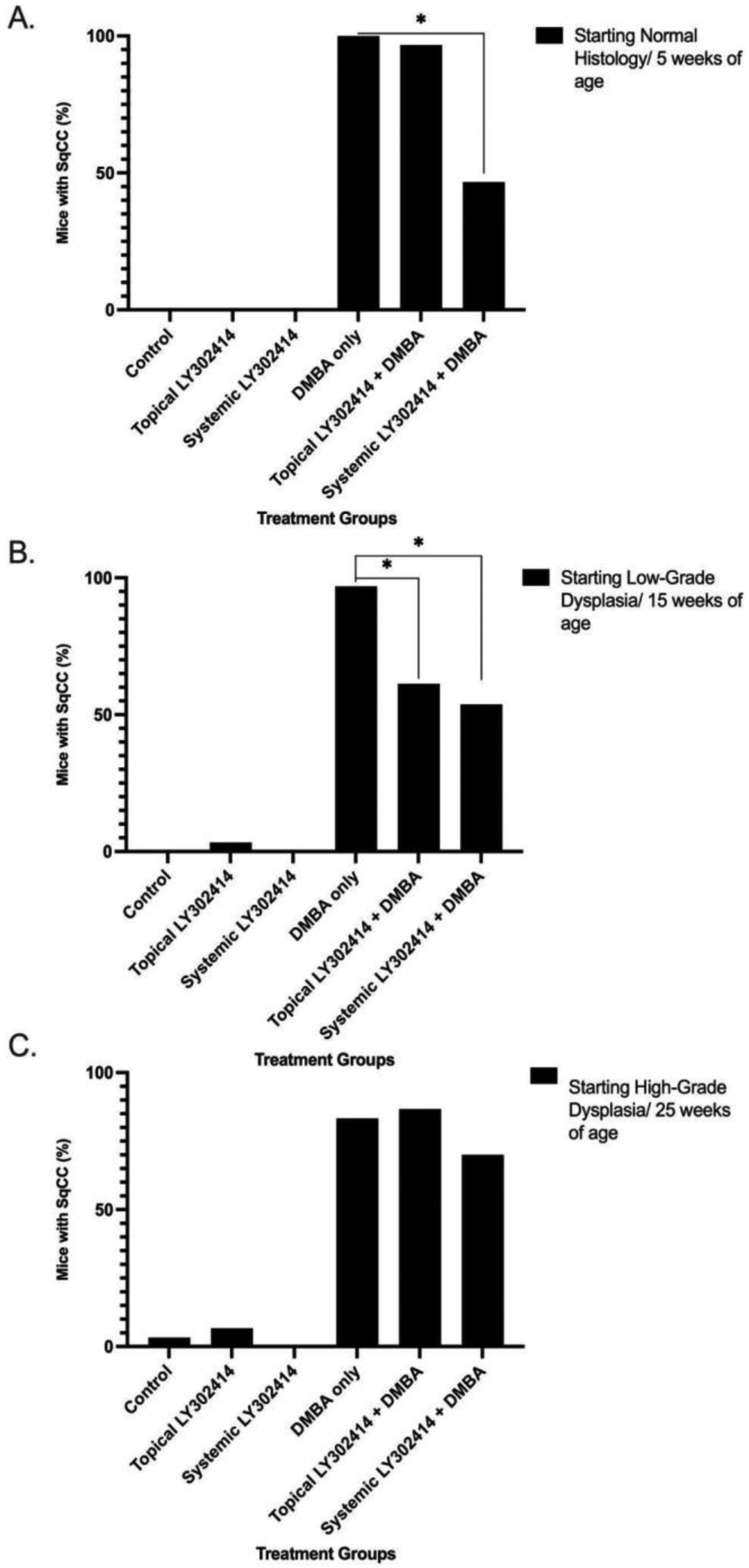
The percentage of *K14E6/E7* mice that developed squamous cell carcinoma (SqCC) of the anus in each treatment group at the end of the 20-week treatment period as assessed by histology: H&E-stained anal tissue. The notation * represents statistical significance (p < 0.05) between groups. **A.** Mice that began treatment at 5 weeks of age/ normal anal histology. **B.** Mice that began treatment at 15 weeks of age/ low-grade anal dysplasia. **C.** Mice that began treatment at 25 weeks of age/high-grade anal dysplasia.

None of the control (0/30) or LY3023414-only groups (topical (0/30) or systemic (0/14)) developed histologically-proven anal cancer—squamous cell carcinoma (SqCC) of the anus. All of the mice treated with topical DMBA alone (30/30) developed SqCC of the anus and the vast majority, 96.8% (30/31) of mice given topical LY3023414 in conjunction with topical DMBA developed SqCC. There was a statistically significant reduction in the number of mice that developed SqCC when given systemic LY3023414 + topical DMBA, 47% (7/15), as compared to topical DMBA only mice (30/30) (p-value < 0.0001) (Figure 3A).

#### 3.3.1.2. Low-Grade Anal Dysplasia/15 weeks of age

Development of histologically-proven SqCC was seen in none of the control mice (0/30), one of the mice (1/30) given topical LY3023414 alone, and none of the mice treated with systemic LY3023414 alone (0/12). Of those treated topically with DMBA alone, 96.9% (31/32) of mice developed anal SqCC while 61.3% (19/31) of mice treated with topical LY3023414 in conjunction with topical DMBA developed anal SqCC, resulting in a significant decrease in anal SqCC development (p-value = 0.0005). There was also a significant decrease in anal SqCC development in the systemic LY3023414 + topical DMBA treatment group as compared to the topical DMBA only group, as only 53.8% (7/13) of the mice treated systemically with LY3023414 + topical DMBA developed anal SqCC (p-value = 0.0012) (Figure 3B).

#### 3.3.1.3. High-Grade Anal Dysplasia/25 weeks of age

Histologically-proven SqCC development was found in 3.3% (1/30) of the control mice, 6.67% (2/30) of the mice treated with topical LY3023414 alone, and 0% (0/11) of the mice treated with systemic LY3023414 alone. Topical LY3023414 + topical DMBA saw 86.7% (26/30) of mice develop microscopic SqCC, which was not significantly different than the 83.3% of mice (25/30) that developed SqCC when treated with topical DMBA alone (p-value = 1.0000). Microscopic SqCC incidence was also not significantly different when comparing systemic LY3023414 + topical DMBA mice to the topical DMBA only mice, with 70% (7/10) of the systemic LY3023414 + topical DMBA mice progressing to SqCC (p-value = 0.3878) (Figure 3C).

### 3.4 LY3023414 Inhibition of pAKT and pS6 Expression varied with Starting Histology

#### 3.4.1. Normal Histology/5 weeks of age

**Figure 4:**
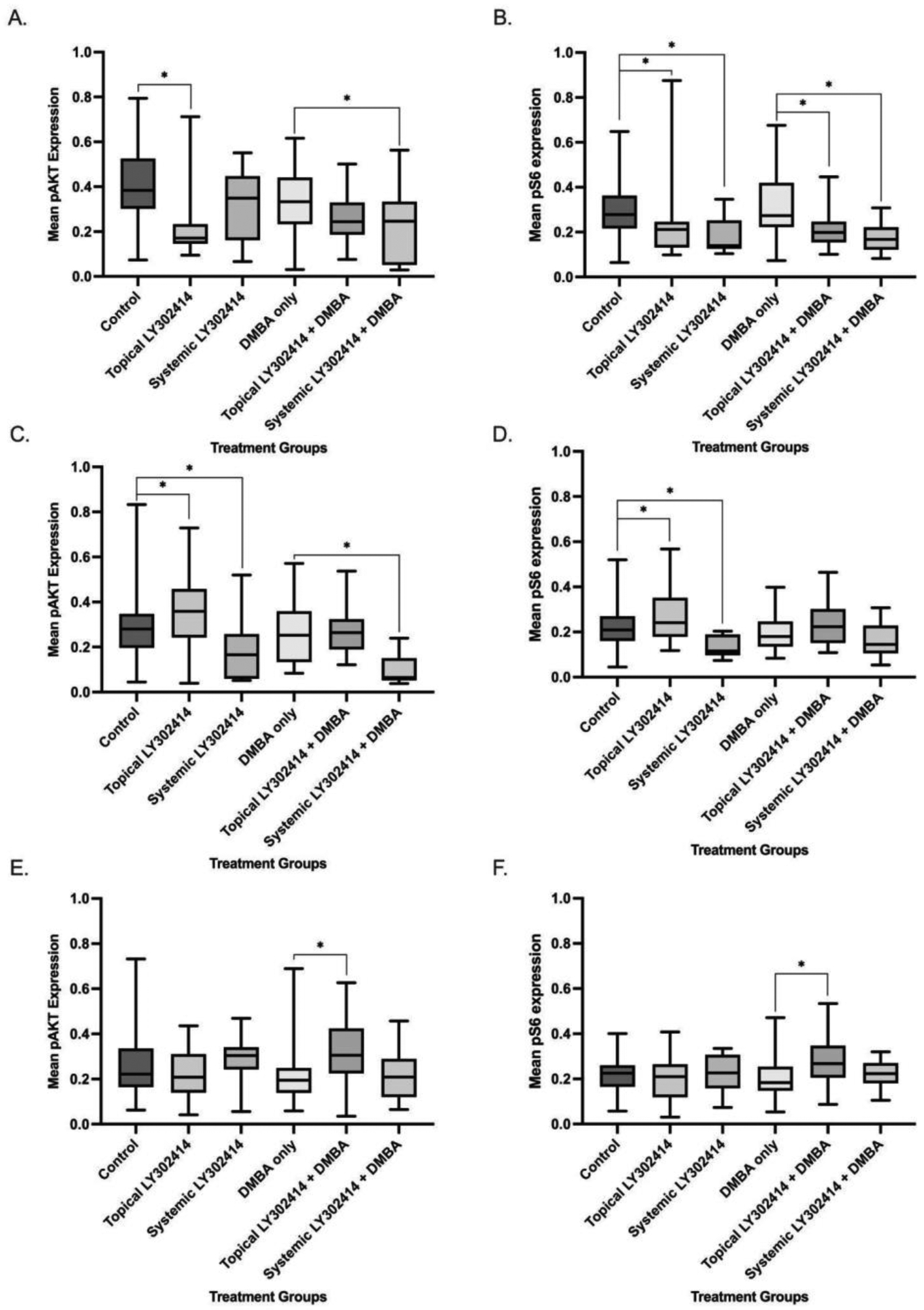
Mean pAKT and pS6 protein expression, markers of PI3K and mTOR activity respectively, quantified via immunohistochemical staining sections of K14E6/E7 mice anal tissue. Mean ± standard deviation values are reported in the RawIntDen/Area (raw integrated density/area). The notation * represents statistical significance (p < 0.05). A, B. Mice that began treatment at 5 weeks of age/ normal anal histology. C, D. Mice that began treatment at 15 weeks of age/ low-grade anal dysplasia. E, F. Mice that began treatment at 25 weeks of age/ high-grade anal dysplasia.

Immunohistochemistry for pAKT was performed to assess the target effects of PI3K. Mice had significant reductions in pAKT expression when treated with topical LY3023414 alone (mean value = 0.235 ± 0.161) as compared to the control (mean value = 0.410 ± 0.162; p-value < 0.0001). This significant reduction in pAKT expression indicated inhibition of the PI3K pathway. When compared to the control group, the systemic LY3023414-only treatment group (mean value = 0.311 ± 0.171) did not have a significant reduction in pAKT expression (p-value = 0.0696). The topical LY3023414 + topical DMBA treatment group (mean value = 0.259 ± 0.106), as compared to the topical DMBA-only treatment group (mean value = 0.337 ± 0.149), did not have a significant decrease in pAKT expression (p-value = 0.1237). There was a significant decrease in pAKT expression–indicating inhibition of the PI3K pathway–when comparing the systemic LY3023414 + topical DMBA treatment group (mean value = 0.238 ± 0.159) to the topical DMBA-only treatment group (p-value = 0.0473) (Figure 4A).

The target effect of mTOR was assessed via immunohistochemical staining for pS6. The topical LY3023414-only (mean value = 0.241 ± 0.169) and systemic LY3023414-only (mean value = 0.181 ± 0.080) treatment groups significantly decreased in pS6 expression when compared to the control group (mean value = 0.296 ± 0.123; p-values = 0.0199 and 0.0027, respectively). There were also significant reductions in expression of pS6 in both the topical LY3023414 + topical DMBA treatment group (mean value = 0.211 ± 0.081) and systemic LY3023414 + topical DMBA treatment group (mean value = 0.175 ± 0.068) as compared to the topical DMBA-only group (mean value = 0.313 ± 0.138; p-values = 0.0015 and 0.0008, respectively). The significant reductions in pS6 expression indicate inhibition of the mTOR pathway (Figure 4B).

#### 3.4.2. Low-Grade Anal Dysplasia/15 weeks of age

There was a significant increase in pAKT expression seen in the topical LY3023414-only tissue (mean value = 0.365 ± 0.160) while a significant decrease in pAKT expression was seen in systemic LY3023414 only (mean value = 0.180 ± 0.139) when compared to control (mean value = 0.291 ± 0.150; p-values = 0.0492 and 0.0150, respectively). Of the mice treated topically with LY3023414 + topical DMBA mice, there was no significant difference seen in pAKT expression (mean value = 0.270 ± 0.101) when compared to topical DMBA-only tissue (mean value = 0.253 ± 0.123; p-value = 0.3530). However, a significant decrease in pAKT expression was seen in the systemically-treated LY3023414 + topical DMBA treatment group (mean value = 0.098 ± 0.070) as compared to DMBA only (mean value = 0.253 ± 0.123; p-value = 0.0005) indicating inhibition of the PI3K pathway (Figure 4C). Comparable to pAKT expression, a significant increase in pS6 expression was seen in the mice treated with topical LY3023414 alone (mean value = 0.270 ± 0.111) while the systemically-treated LY3023414-only group had a significant decrease in pS6 expression (mean value = 0.132 ± 0.048) when compared to the control group (mean value = 0.224 ± 0.098; p-values = 0.0482 and 0.0018, respectively). No significant differences in pS6 expression were seen in the topical LY3023414 + topical DMBA (mean value = 0.233 ± 0.102) or the systemic LY3023414 + topical DMBA (mean value = 0.168 ± 0.074) treatment groups when compared to the topical DMBA-only group (mean value = 0.195 ± 0.081; p-values = 0.1061 and 0.3170, respectively) (Figure 4D).

#### 3.4.3. High-Grade Anal Dysplasia/25 weeks of age

No significant difference in pAKT expression was seen in mice treated with topical LY3023414 alone (mean value = 0.220 ± 0.104) or systemic LY3023414 alone (mean value = 0.300 ± 0.110) when compared to the control mice (mean value = 0.261 ± 0.146; p-values = 0.1685 and 0.4332, respectively). There was a significant increase in pAKT expression in mice treated with topical LY3023414 + topical DMBA (mean value = 0.319 ± 0.136) compared to the topical DMBA-only group (mean value = 0.227 ± 0.143; p-value = 0.0020). There was no change in pAKT in the systemic LY3023414 + topical DMBA group (mean value = 0.216 ± 0.120) compared to topical DMBA-only group (p-value = 0.8359) (Figure 4E). Neither the topical LY3023414-only group (mean value = 0.191 ± 0.090) nor the systemic LY3023414-only (mean value = 0.220 ± 0.084) significantly differed in pS6 expression when compared to the control group (mean value = 0.220 ± 0.075; p-values = 0.1732 and 0.9920, respectively). Topical LY3023414 + topical DMBA significantly increased pS6 expression (mean value = 0.273 ± 0.099) compared to the topical DMBA-only group (mean value = 0.211 ± 0.092; p-value = 0.0032). There was also no difference in pS6 expression between mice treated with systemic LY3023414 + topical DMBA (mean value = 0.221 ± 0.068) compared to the topical DMBA-only group (p-value = 0.7457) (Figure 4F).

## 4. Discussion

Anal dysplasia and anal cancer are increasing problems nationally [1]. Recent results from the ANal Cancer/HSIL Outcomes Research (ANCHOR) trial have shown that treatment of anal dysplasia has the capacity to decrease anal cancer development [28]. These findings support the treatment of precancerous anal lesions for anal cancer prevention. As stated above, current treatments for anal dysplasia are not targeted to HPV-infected cells and thus cause toxicity to uninfected cells and local side effects. To target therapies against HPV, it is important to review which pathways are affected by the virus. Upon HPV infection, E6 and E7 oncoprotein expression alters pathways that are essential to carcinogenesis, including the PI3K and mTOR pathways. Thus, targeting these pathways is one way to target HPV infected cells more directly. Route of administration of drug therapy is based on convenience, compliance, pharmacokinetics, and pharmacodynamic profile. Oral administration is convenient, cost effective, and the most commonly used route of administration of drugs (Ruiz & Scioli). However, oral drug administration leads to high systemic drug concentrations and thus increased potential for drug-drug interactions. A topical, anal/rectal route allows for rapid and effective absorption of drugs at the area of concern for anal dysplasia and decreased systemic effects. However, topical drug application often decreases patient compliance. In this study, we evaluated two modes of drug delivery, topical and systemic, to determine whether either method was more efficacious. Our results show that systemic LY3023414 treatment was overall more effective than topical therapy, in terms of preventing overt anal tumor development, increasing the rate of tumor-free survival, and reducing histological evidence of cancer in mice that began treatment with normal anal histology or low-grade dysplasia of the anus. The fact that systemic mode of delivery was found to be more beneficial than topical may be related to the substrate used to dilute the LY3023414. In order to provide the most benefit in a topical therapy, a viscous diluent should be used to increase the duration of drug contact to the tissue of interest. Polyethylene glycol (PEG-300), though viscous, may not provide the ideal contact duration for long-term use. This theory is corroborated by the greater inhibition of the PI3K (pAKT) and mTOR (pS6) expression seen in mice starting with normal histology or low-grade anal dysplasia that received systemic therapy as opposed to topical therapy. In future studies, concentrations of target drugs in the mice anal tissue will be measured to ensure proper absorption at the anus.

Throughout anal carcinogenesis, there are changes that occur in PI3K/mTOR pathway inhibition and activation at various degrees of dysplasia [26]. For this reason, we evaluated the effects of LY3023414 treatment at both low and high grades of anal disease as well as normal anal histology. Our findings support the use of a dual PI3K/mTOR inhibitor via topical and systemic modes of delivery to prevent cancer progression to tumors in mice starting with normal histology and low-grade anal dysplasia. In contrast, LY3023414 produced a deleterious effect on mice displaying high-grade anal dysplasia; there was a slight increase in carcinoma development in mice that were treated with topical LY3023414. The difference in outcome for mice with low-grade versus high grade anal dysplasia likely reflects the changes that occur in PI3K and mTOR pathways activation throughout the process of anal carcinogenesis. In addition, LY3023414 did not effectively inhibit pAKT or pS6 expression in mice that began treatment with starting high-grade anal dysplasia. In fact, protein expression tended to increase with LY3023414 application, highlighting an unexpected response to the pathway inhibitor. Limitations to this study include having an unequal number of mice in each treatment group. Initial groups of mice were filled to properly power the study with a minimum of 12 mice per group. Not surprisingly, a few older mice from the high-grade dysplastic treatment groups died before the end of treatment duration, due to non-drug related health issues. Extra mice were added to later treatment groups to compensate for the loss of mice. Another limitation of this study is the lack of LY3023414 quantification in both the mice anal tissue and mice sera. The small size of mouse anal tissue often poses a challenge when preparing tissue for histology and portioning for sample absorption analysis. We have included immunohistochemical data that has significantly more variation than tissue concentration data, as seen in the standard deviation of the intensity signal.

## 5. Conclusions

Both treatment modes, topical and systemic, are effective in reducing and delaying anal tumor development in HPV-associated-mice starting with normal anal histology or low-grade anal dysplasia. Systemic PI3K/mTOR inhibition via LY3023414 is more effective than topical application in preventing anal SqCC in mice with normal anal histology or low-grade anal dysplasia. The use of LY3023414 is not beneficial in the treatment of high-grade dysplasia to prevent anal cancer.

## Abbreviations:

5-FU: 5-Fluorouracil
ANCHOR: ANal Cancer/HSIL Outcomes Research
DAB: 3,3’-Diaminobenzidine
DMBA: 7,12 dimethylbenz [a] anthracene, DMSO: Dimethyl Sulfoxide
HE: Hematoxylin and Eosin
HCl: Hydrochloric Acid
HIV: Human Immunodeficiency Virus
HPV: Human Papillomavirus
mTOR: Mammalian (mechanistic) target of Rapamycin
mTORC1: Mammalian (mechanistic) Target of Rapamycin Complex 1
mTORC2: Mammalian (mechanistic) target of rapamycin complex 2
pAKT: Phosphorylated AKT Serine/Threonine Kinase 1
PBS: Phosphate Buffered Saline
PEG: Polyethylene Glycol
PI3K: Phosphoinositide 3 Kinase
pS6: Phosphorylated S6
RawIntDen/Area: Raw Integrated Density/Area
SqCC: Squamous Cell Carcinoma
